# (5-Meth­oxy-1*H*-indol-3-yl)acetonitrile

**DOI:** 10.1107/S1600536811051762

**Published:** 2011-12-10

**Authors:** Yu-Hua Ge, Jin-Feng Li, Yang-Hui Luo

**Affiliations:** a Ordered Matter Science Research Center, College of Chemistry and Chemical Engineering, Southeast University, Nanjing 210096, People’s Republic of China

## Abstract

In the title compound, C_11_H_10_N_2_O, the O atom and the C atom of the methyl­ene group deviate only slightly [0.029 (3) and 0.055 (3) Å, respectively] from the approximately planar ring system (r.m.s. deviation = 0.013 Å). In the crystal, N—H⋯O hydrogen bonds link the mol­ecules into zigzag chains running along the *b* axis.

## Related literature

Indole-3-acetic acid is recognized as the key auxin in most plants, see:  see: Woodward & Bartel (2005 ▶).
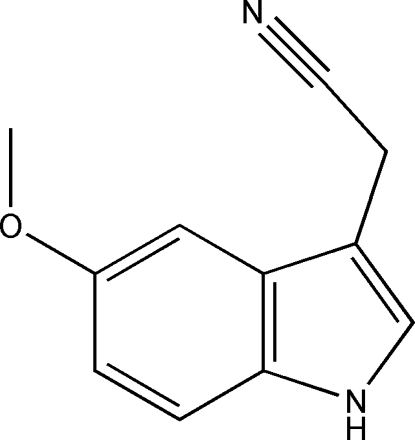


## Experimental

### 

#### Crystal data

C_11_H_10_N_2_O
*M* *_r_* = 186.21
Monoclinic, 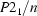

*a* = 8.9242 (18) Å
*b* = 11.461 (2) Å
*c* = 9.889 (2) Å
β = 110.77 (3)°
*V* = 945.7 (3) Å^3^
*Z* = 4
Mo *K*α radiation
μ = 0.09 mm^−1^
*T* = 293 K
0.20 × 0.20 × 0.20 mm

#### Data collection

Rigaku SCXmini diffractometer
Absorption correction: multi-scan (*CrystalClear*; Rigaku, 2005 ▶) *T*_min_ = 0.983, *T*_max_ = 0.983
9617 measured reflections
2164 independent reflections
1225 reflections with *I* > 2σ(*I*)
*R*_int_ = 0.085

#### Refinement

*R*[*F* ^2^ > 2σ(*F* ^2^)] = 0.064
*wR*(*F* ^2^) = 0.162
*S* = 1.01
2164 reflections
127 parameters
H-atom parameters constrained
Δρ_max_ = 0.16 e Å^−3^
Δρ_min_ = −0.23 e Å^−3^

### 

Data collection: *CrystalClear* (Rigaku, 2005 ▶); cell refinement: *CrystalClear*; data reduction: *CrystalClear*; program(s) used to solve structure: *SHELXS97* (Sheldrick, 2008 ▶); program(s) used to refine structure: *SHELXL97* (Sheldrick, 2008 ▶); molecular graphics: *DIAMOND* (Brandenburg & Putz, 2005 ▶); software used to prepare material for publication: *SHELXL97*.

## Supplementary Material

Crystal structure: contains datablock(s) I, global. DOI: 10.1107/S1600536811051762/bt5708sup1.cif

Structure factors: contains datablock(s) I. DOI: 10.1107/S1600536811051762/bt5708Isup2.hkl

Supplementary material file. DOI: 10.1107/S1600536811051762/bt5708Isup3.cml

Additional supplementary materials:  crystallographic information; 3D view; checkCIF report

## Figures and Tables

**Table 1:** Hydrogen-bond geometry (Å, °)

*D*—H⋯*A*	*D*—H	H⋯*A*	*D*⋯*A*	*D*—H⋯*A*
N1—H1*A*⋯O1^i^	0.86	2.18	3.038 (3)	175

Symmetry code: (i) 
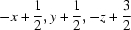
.

**Fig. 1.**
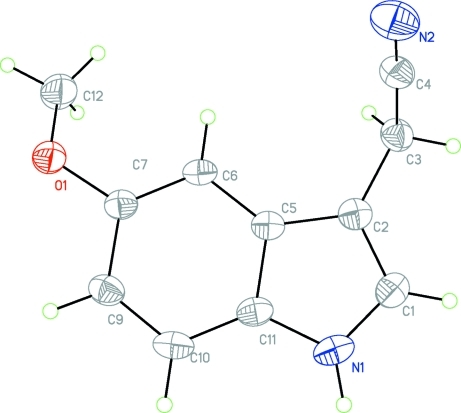
The molecular structure of the title compound with the atom-labelling scheme. Displacement ellipsoids are drawn at the 30% probability level. H atoms are represented as small spheres of arbitrary radii.

**Fig. 2.**
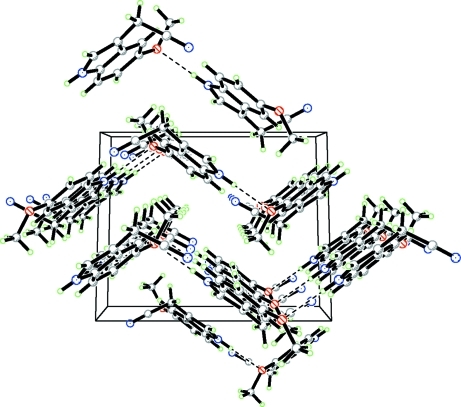
A packing view down the a axis showing the three dimensionnal network. Intermolecular hydrogen bonds are shown as dashed lines.

## supplementary crystallographic information

### Comment

The derivatives of indole are important chemical materials, because they are excellent drug intermediates for many pharmaceutical products. As part of our interest in these materials, we report here the crystal structure of the title compound.

The molecular structure of the title compound is shown in Fig. 1. The atoms O1 and N3 are located in the indole plane. The title compound formed zigzag chain structure *via* intermolecular N—H···O hydrogen bonds interactions (Fig. 2).

### Experimental

Crystals of 3-cyano-5-methoxyindole suitable for X-ray diffraction were obstained by slow evaporation of a methanol solution.

### Refinement

All H atoms attached to C atoms and N atoms were fixed geometrically and treated as riding with C—H = 0.93 Å (CH), C—H = 0.97 Å (CH_2_), C—H = 0.96 Å (CH_3_)and N—H = 0.86 Å with *U*_iso_(H) = 1.2*U*_eq_(CH, CH_2_ and NH) and *U*_iso_(H) = 1.5*U*_eq_(CH_3_).

### Crystal data

**Table:**

C_11_H_10_N_2_O	*F*(000) = 392
*M**_r_* = 186.21	*D*_x_ = 1.308 Mg m^−^^3^
Monoclinic, *P*2_1_/*n*	Mo *K*α radiation, λ = 0.71073 Å
Hall symbol: -P 2yn	Cell parameters from 2164 reflections
*a* = 8.9242 (18) Å	θ = 3.0–27.5°
*b* = 11.461 (2) Å	µ = 0.09 mm^−^^1^
*c* = 9.889 (2) Å	*T* = 293 K
β = 110.77 (3)°	Prism, white
*V* = 945.7 (3) Å^3^	0.20 × 0.20 × 0.20 mm
*Z* = 4	

### Data collection

**Table:**

Rigaku SCXmini diffractometer	2164 independent reflections
Radiation source: fine-focus sealed tube	1225 reflections with *I* > 2σ(*I*)
graphite	*R*_int_ = 0.085
Detector resolution: 13.6612 pixels mm^-1^	θ_max_ = 27.5°, θ_min_ = 3.0°
CCD_Profile_fitting scans	*h* = −11→11
Absorption correction: multi-scan (*CrystalClear*; Rigaku, 2005)	*k* = −14→14
*T*_min_ = 0.983, *T*_max_ = 0.983	*l* = −12→12
9617 measured reflections	

### Refinement

**Table:**

Refinement on *F*^2^	Primary atom site location: structure-invariant direct methods
Least-squares matrix: full	Secondary atom site location: difference Fourier map
*R*[*F*^2^ > 2σ(*F*^2^)] = 0.064	Hydrogen site location: inferred from neighbouring sites
*wR*(*F*^2^) = 0.162	H-atom parameters constrained
*S* = 1.01	*w* = 1/[σ^2^(*F*_o_^2^) + (0.0742*P*)^2^] where *P* = (*F*_o_^2^ + 2*F*_c_^2^)/3
2164 reflections	(Δ/σ)_max_ < 0.001
127 parameters	Δρ_max_ = 0.16 e Å^−^^3^
0 restraints	Δρ_min_ = −0.23 e Å^−^^3^

### Special details

**Table:**

Geometry. All e.s.d.'s (except the e.s.d. in the dihedral angle between two l.s. planes) are estimated using the full covariance matrix. The cell e.s.d.'s are taken into account individually in the estimation of e.s.d.'s in distances, angles and torsion angles; correlations between e.s.d.'s in cell parameters are only used when they are defined by crystal symmetry. An approximate (isotropic) treatment of cell e.s.d.'s is used for estimating e.s.d.'s involving l.s. planes.
Refinement. Refinement of *F*^2^ against ALL reflections. The weighted *R*-factor *wR* and goodness of fit *S* are based on *F*^2^, conventional *R*-factors *R* are based on *F*, with *F* set to zero for negative *F*^2^. The threshold expression of *F*^2^ > σ(*F*^2^) is used only for calculating *R*-factors(gt) *etc*. and is not relevant to the choice of reflections for refinement. *R*-factors based on *F*^2^ are statistically about twice as large as those based on *F*, and *R*- factors based on ALL data will be even larger.

### Fractional atomic coordinates and isotropic or equivalent isotropic displacement parameters (Å^2^)

**Table:**

	*x*	*y*	*z*	*U*_iso_*/*U*_eq_	
O1	0.08200 (19)	0.23891 (13)	0.92113 (17)	0.0519 (5)	
N1	0.4881 (3)	0.52233 (16)	0.7682 (2)	0.0518 (6)	
H1A	0.4745	0.5834	0.7146	0.062*	
C11	0.3678 (3)	0.45969 (19)	0.7916 (2)	0.0427 (6)	
C7	0.1867 (3)	0.30957 (19)	0.8827 (2)	0.0411 (6)	
C6	0.3486 (3)	0.29199 (18)	0.9310 (2)	0.0397 (6)	
H6A	0.3953	0.2306	0.9932	0.048*	
C5	0.4415 (3)	0.36821 (17)	0.8848 (2)	0.0375 (5)	
C9	0.1145 (3)	0.40038 (19)	0.7890 (2)	0.0471 (6)	
H9A	0.0039	0.4097	0.7578	0.056*	
C2	0.6091 (3)	0.37808 (19)	0.9158 (2)	0.0435 (6)	
C10	0.2039 (3)	0.4759 (2)	0.7425 (2)	0.0493 (7)	
H10A	0.1559	0.5366	0.6795	0.059*	
C4	0.7188 (3)	0.1836 (2)	0.9369 (3)	0.0512 (7)	
N2	0.7006 (3)	0.0969 (2)	0.8796 (3)	0.0763 (8)	
C3	0.7349 (3)	0.2968 (2)	1.0056 (3)	0.0499 (7)	
H3A	0.8399	0.3288	1.0192	0.060*	
H3B	0.7258	0.2884	1.1000	0.060*	
C1	0.6310 (3)	0.4726 (2)	0.8428 (2)	0.0488 (6)	
H1B	0.7297	0.4993	0.8438	0.059*	
C12	0.1430 (3)	0.1826 (2)	1.0567 (3)	0.0674 (8)	
H12A	0.0603	0.1361	1.0707	0.101*	
H12B	0.1792	0.2401	1.1319	0.101*	
H12C	0.2312	0.1334	1.0596	0.101*	

### Atomic displacement parameters (Å^2^)

**Table:**

	*U*^11^	*U*^22^	*U*^33^	*U*^12^	*U*^13^	*U*^23^
O1	0.0497 (10)	0.0531 (11)	0.0501 (10)	−0.0009 (8)	0.0141 (9)	0.0083 (8)
N1	0.0763 (15)	0.0387 (11)	0.0433 (11)	−0.0030 (11)	0.0248 (11)	0.0039 (9)
C11	0.0580 (15)	0.0334 (13)	0.0369 (12)	0.0000 (11)	0.0171 (12)	−0.0018 (10)
C7	0.0483 (14)	0.0359 (13)	0.0375 (12)	−0.0007 (10)	0.0132 (11)	−0.0029 (10)
C6	0.0510 (15)	0.0296 (12)	0.0354 (12)	0.0022 (10)	0.0116 (11)	0.0016 (9)
C5	0.0482 (14)	0.0336 (12)	0.0297 (11)	0.0016 (10)	0.0127 (10)	−0.0052 (9)
C9	0.0496 (15)	0.0413 (14)	0.0422 (13)	0.0042 (11)	0.0063 (12)	−0.0013 (11)
C2	0.0533 (15)	0.0397 (14)	0.0387 (13)	0.0006 (11)	0.0181 (12)	−0.0064 (10)
C10	0.0649 (17)	0.0367 (14)	0.0393 (13)	0.0084 (12)	0.0098 (13)	0.0051 (10)
C4	0.0540 (16)	0.0443 (16)	0.0596 (16)	0.0075 (12)	0.0253 (13)	0.0017 (13)
N2	0.0910 (19)	0.0520 (16)	0.0877 (18)	0.0086 (13)	0.0337 (15)	−0.0115 (14)
C3	0.0490 (14)	0.0445 (15)	0.0569 (15)	0.0016 (11)	0.0197 (12)	−0.0065 (12)
C1	0.0589 (16)	0.0443 (15)	0.0497 (14)	−0.0050 (12)	0.0274 (13)	−0.0078 (11)
C12	0.0650 (18)	0.0741 (19)	0.0598 (17)	−0.0023 (15)	0.0180 (14)	0.0212 (15)

### Geometric parameters (Å, °)

**Table:**

O1—C7	1.387 (3)	C9—H9A	0.9300
O1—C12	1.411 (3)	C2—C1	1.353 (3)
N1—C1	1.352 (3)	C2—C3	1.487 (3)
N1—C11	1.378 (3)	C10—H10A	0.9300
N1—H1A	0.8600	C4—N2	1.127 (3)
C11—C5	1.397 (3)	C4—C3	1.448 (3)
C11—C10	1.381 (3)	C3—H3A	0.9700
C7—C6	1.366 (3)	C3—H3B	0.9700
C7—C9	1.391 (3)	C1—H1B	0.9300
C6—C5	1.389 (3)	C12—H12A	0.9600
C6—H6A	0.9300	C12—H12B	0.9600
C5—C2	1.420 (3)	C12—H12C	0.9600
C9—C10	1.363 (3)		
			
C7—O1—C12	117.14 (18)	C5—C2—C3	126.4 (2)
C1—N1—C11	109.2 (2)	C9—C10—C11	118.0 (2)
C1—N1—H1A	125.4	C9—C10—H10A	121.0
C11—N1—H1A	125.4	C11—C10—H10A	121.0
N1—C11—C5	106.8 (2)	N2—C4—C3	177.3 (3)
N1—C11—C10	131.5 (2)	C4—C3—C2	110.65 (19)
C5—C11—C10	121.7 (2)	C4—C3—H3A	109.5
C6—C7—O1	123.33 (19)	C2—C3—H3A	109.5
C6—C7—C9	121.7 (2)	C4—C3—H3B	109.5
O1—C7—C9	114.9 (2)	C2—C3—H3B	109.5
C7—C6—C5	118.2 (2)	H3A—C3—H3B	108.1
C7—C6—H6A	120.9	C2—C1—N1	110.0 (2)
C5—C6—H6A	120.9	C2—C1—H1B	125.0
C6—C5—C11	119.5 (2)	N1—C1—H1B	125.0
C6—C5—C2	133.2 (2)	O1—C12—H12A	109.5
C11—C5—C2	107.2 (2)	O1—C12—H12B	109.5
C10—C9—C7	120.8 (2)	H12A—C12—H12B	109.5
C10—C9—H9A	119.6	O1—C12—H12C	109.5
C7—C9—H9A	119.6	H12A—C12—H12C	109.5
C1—C2—C5	106.8 (2)	H12B—C12—H12C	109.5
C1—C2—C3	126.8 (2)		

### Hydrogen-bond geometry (Å, °)

**Table:**

*D*—H···*A*	*D*—H	H···*A*	*D*···*A*	*D*—H···*A*
N1—H1A···O1^i^	0.86	2.18	3.038 (3)	175.

Symmetry codes: (i) −*x*+1/2, *y*+1/2, −*z*+3/2.

## References

[bb1] Brandenburg, K. & Putz, H. (2005). *DIAMOND* Crystal Impact GbR, Bonn, Germany.

[bb2] Rigaku. (2005). *CrystalClear* Rigaku Corporation, Tokyo, Japan.

[bb3] Sheldrick, G. M. (2008). *Acta Cryst.* A**64**, 112–122. 10.1107/S0108767307043930 18156677

[bb4] Woodward, A. W. & Bartel, B. (2005). *Ann. Bot.* **95**, 707–735. 10.1093/aob/mci083 PMC4246732 15749753

